# Ethnic minority representation in UK COVID-19 trials: systematic review and meta-analysis

**DOI:** 10.1186/s12916-023-02809-7

**Published:** 2023-03-29

**Authors:** Mayur Murali, Leher Gumber, Hannah Jethwa, Divolka Ganesh, Jamie Hartmann-Boyce, Harpreet Sood, Francesco Zaccardi, Kamlesh Khunti

**Affiliations:** 1 Division of Anaesthetics, Pain Medicine & Intensive Care, Department of Surgery & Cancer, Faculty of Medicine, Imperial College London, London, UK; 2 Northumbria Healthcare NHS Foundation Trust, Northumberland, UK; 3 London North West University Healthcare NHS Trust, London, UK; 4 Leeds Teaching Hospitals Trust, West Yorkshire, UK; 5 Nuffield Department of Primary Care Health Sciences, University of Oxford, Oxford, UK; 6 Health Education England, London, UK; 7 Diabetes Research Centre, University of Leicester, Leicester, UK

**Keywords:** Ethnicity, COVID-19, Medical research, Clinical trials

## Abstract

**Background:**

The COVID-19 pandemic has highlighted health disparities affecting ethnic minority communities. There is growing concern about the lack of diversity in clinical trials. This study aimed to assess the representation of ethnic groups in UK-based COVID-19 randomised controlled trials (RCTs).

**Methods:**

A systematic review and meta-analysis were undertaken. A search strategy was developed for MEDLINE (Ovid) and Google Scholar (1st January 2020–4th May 2022). Prospective COVID-19 RCTs for vaccines or therapeutics that reported UK data separately with a minimum of 50 participants were eligible. Search results were independently screened, and data extracted into proforma. Percentage of ethnic groups at all trial stages was mapped against Office of National Statistics (ONS) statistics. Post hoc DerSimonian-Laird random-effects meta-analysis of percentages and a meta-regression assessing recruitment over time were conducted. Due to the nature of the review question, risk of bias was not assessed. Data analysis was conducted in Stata v17.0. A protocol was registered (PROSPERO CRD42021244185).

**Results:**

In total, 5319 articles were identified; 30 studies were included, with 118,912 participants. Enrolment to trials was the only stage consistently reported (17 trials). Meta-analysis showed significant heterogeneity across studies, in relation to census-expected proportions at study enrolment. All ethnic groups, apart from Other (1.7% [95% CI 1.1–2.8%] vs ONS 1%) were represented to a lesser extent than ONS statistics, most marked in Black (1% [0.6–1.5%] vs 3.3%) and Asian (5.8% [4.4–7.6%] vs 7.5%) groups, but also apparent in White (84.8% [81.6–87.5%] vs 86%) and Mixed 1.6% [1.2–2.1%] vs 2.2%) groups. Meta-regression showed recruitment of Black participants increased over time (*p* = 0.009).

**Conclusions:**

Asian, Black and Mixed ethnic groups are under-represented or incorrectly classified in UK COVID-19 RCTs. Reporting by ethnicity lacks consistency and transparency. Under-representation in clinical trials occurs at multiple levels and requires complex solutions, which should be considered throughout trial conduct. These findings may not apply outside of the UK setting.

**Supplementary Information:**

The online version contains supplementary material available at 10.1186/s12916-023-02809-7.

## Background

Since the emergence of the Coronavirus disease (COVID-19) pandemic in January 2020 in the United Kingdom (UK), longstanding health disparities affecting ethnic minority communities have come to light [1]. Emerging evidence has shown that ethnic minorities have had the highest rate of diagnosis [2], severe disease requiring advanced respiratory support [3] and mortality [4–6]. Several reasons for the observed differences have been proposed including higher rates of social deprivation; higher rates of pre-existing health conditions (for example type 2 diabetes and cardiovascular disease); greater frequency of living in large or multi-generational households; and poorer access to health services [7–11]. To combat severe acute respiratory distress syndrome 2 (SARS-CoV-2) and limit its transmission and complications, many randomised controlled trials (RCTs) have been conducted globally to determine effective treatments and develop vaccines which have been subsequently rolled out at a population level. Landmark trials have provided compelling evidence for several vaccines such as BNT162b2 messenger ribonucleic acid (mRNA) [12], ChAdOx1 nCoV-19 (AZD1222) [13] and mRNA-1273 [14], and therapies including dexamethasone [15] and sotrovimab [16].

A well-designed and conducted RCT is considered the gold standard (Level I) to evaluate the causal effect of medical interventions. Like other study types, RCTs also depend upon participation of all groups to improve generalisability and validity of the findings. There is growing concern about the lack of diversity in trials across health and clinical research over the last few years [17–20]. This may stem from anxieties around the implications of participation within ethnic minority communities, added costs of participation (such as travel and parking), language barriers, knowledge gaps and lack of diversity within the research team [21–24].

Given the disproportionate impact of COVID-19 on minority individuals, inclusion of ethnic minority populations in COVID-19 trials is vital to understanding differences of interventions in disease severity and outcomes as well as addressing critical gaps in knowledge. Therefore, this systematic review aimed to assess the representation of different ethnic groups in UK-based COVID-19 vaccine and therapeutic trials and compared them to nationally available data on ethnic minority populations in the UK.

## Methods

This systematic review and meta-analysis was conducted and reported in accordance with the Preferred Reporting Items for Systematic Review and Meta-Analyses (PRISMA) guidelines [25] (Supplementary Materials, Appendix 3 and 4). A protocol was registered in advance with the International Prospective Register of Systematic Reviews (PROSPERO, CRD42021244185). Ethical approval was not required.

### Search strategy and selection criteria

A search strategy was developed to identify COVID-19 RCTs that were published in MEDLINE (Ovid) and Google Scholar (Supplementary Materials, Appendix 1). This deviated from the search strategy described in the protocol but was considered comprehensive following discussion with a subject librarian and the review team. We included articles available in English, published between 1st January 2020 and 4th May 2022 in peer-reviewed journals. RCTs were included if they (1) explored COVID-19 vaccine or therapies (medical devices and treatments), (2) were conducted in the UK or reported data separately for the UK population and (3) had a minimum sample size of 50 adults (Table 1). The minimum sample size of 50 patients was a pragmatic decision taken by the authors. We restricted to UK-based studies due to the diversity of the population and good quality ethnicity data at the population level.

**Table 1:** A summary of the inclusion criteria for the meta-analysis and meta-regression

PICOS criteria	Inclusion criteria
Population	Adults ≥ 18 years
Intervention	COVID-19 vaccine or therapeutic treatment
Comparator	Any or none
Outcomes	Any
Study design	Randomised controlled trial (any phase) with a minimum sample size of 50 and conducted in UK or reporting UK data
Time period	Published in peer-reviewed journal between 1st January 2020 and 4th May 2022

Our main outcome was percentage of each ethnic group at different trial stages, for the following: people approached for inclusion; people screened for inclusion; people determined eligible for inclusion; people determined ineligible for inclusion; people enrolled in the trial; people followed up at primary endpoint; people followed up at longest follow-up. However, on review of the data, it became apparent that we could only assess the percentage of ethnic groups enrolled in the included trials due to lack of data availability, though we could report on the number of trials that reported proportions of ethnic groups at each trial stage. We also investigated the number of trials reporting effect estimates by ethnicity at each trial stage as a secondary outcome.

Search results were saved to Rayyan (Qatar Computing Research Institute), a systematic review web-based application. Abstracts were independently screened for inclusion by four review authors (M.M, L.G, H.J and D.G). An online discussion was held between the authors to compare results and adjudicate any discrepancies. Where discrepancies could not be resolved by discussion, they were referred to a second review author. Following exclusion of studies which did not meet the inclusion criteria and duplicates, full-text screening was carried out independently and in duplicate (by M.M, L.G, H.J and D.G) and data was extracted into piloted proforma. Due to the nature of the review questions, we did not assess risk of bias.

### Data analysis

Data were collected on participant demographics (age and sex), type of intervention (vaccine or treatment), total number of participants and general study characteristics. To assess our main outcome, we extracted additional data on the reporting of ethnic diversity of participants at each stage of the trial. We also documented the approach to recruitment, and whether efforts were made to recruit from ethnic minority communities. We documented the enrolment period for each trial to investigate whether recruitment of ethnic minorities changed over time. All studies which reported any ethnicity data were included in the final analysis.

The percentage of each ethnic group within each trial was calculated as a proportion and mapped against national population statistics using Office of National Statistics (ONS) 2011 data for each outcome.

We initially anticipated that heterogeneity in reporting would preclude statistical synthesis and had planned to only calculate percentage of each ethnic group in each trial and map this against national population statistics using forest plots, as reported in our protocol. However, after data extraction, we found more similarities and detailed reporting than we had anticipated, and hence conducted the following post hoc statistical analyses. A DerSimonian-Laird random-effects meta-analysis and a meta-regression to assess changes in recruitment over time were conducted in Stata v 17.0, following a logit transformation of study-specific nonzero proportions and confidence intervals (obtained with Wilson’s method). The meta-regression was conducted to reflect the change in recruitment practices that may have occurred during the pandemic. A *p* < 0.05 was deemed indicative of statistical significance.

### Role of the funding source

 This study was funded by the South Asian Health Foundation. In addition, it was supported by members (K.K, F.Z) of the National Institute for Health Research (NIHR) Applied Research Collaboration East Midlands (ARC-EM).

## Results

**Fig. 1:**
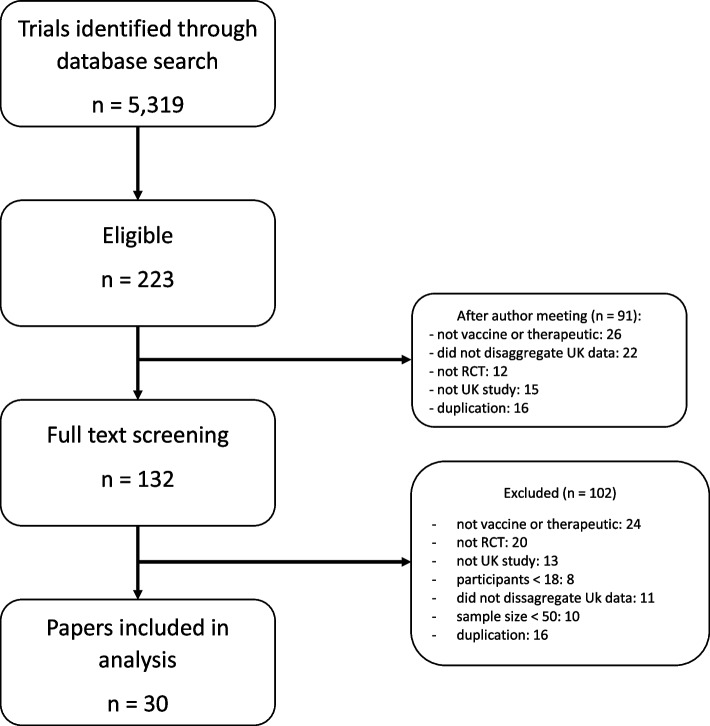
Study selection

A total of 5319 studies were identified through the database search, and 5096 studies were excluded during the abstract screening phase. After removal of duplicates and those that did not meet the inclusion criteria, we reviewed 132 trials for full-text screening, after which a further 102 trials were excluded (Fig. 1) and a total of 30 were included (see Table 2 for a list of identified articles and their characteristics). The most common reasons for exclusion at full text stage were because data were not reported by country, meaning UK data could not be extracted, or the included paper did not assess a COVID-19 vaccine or therapeutic. Of the included studies, 21 were for therapeutic trials and 9 were COVID-19 vaccine trials. One study that met the inclusion criteria did not include any data on ethnicity.

**Table 2:** A total of 30 studies were included in the meta-analysis and meta-regression following a MEDLINE (Ovid) and Google Scholar search

No	Title	Authors	Year	Intervention	Total participants	Mean age	% male	Vaccine/ Therapeutic	Enrolment period
1	Safety and efficacy of NVX-CoV2373 Covid-19 vaccine	Heath PT et al	2021	NVX-CoV2373	14,039	56	51.56%	Vaccine	Sep 2020—Nov 2020
2	Inhaled budesonide for COVID-19 in people at high risk of complications in the community in the UK (PRINCIPLE): a randomised, controlled, open-label, adaptive platform trial	Yu LM et al	2021	Budesonide	3006	64	46.57%	Therapeutic	Nov 2020–Mar 2021
3	Doxycycline for community treatment of suspected COVID-19 in people at high risk of adverse outcomes in the UK: a randomised, controlled, open-label, adaptive platform trial	Butler CC et al	2021	Doxycycline	1792	61	44.16%	Therapeutic	Jul 2020–Dec 2020
4	Azithromycin for community treatment of suspected COVID-19 in people at increased risk of an adverse clinical course in the UK (PRINCIPLE): a randomised, controlled, open-label, adaptive platform trial	Butler CC et al	2021	Azithromycin	1388	60.7	43.22%	Therapeutic	Jul–Nov 2020
5	Safety and efficacy of inhaled nebulised interferon beta-1a (SNG001) for treatment of SARS-CoV-2 infection: a randomised, double-blind, placebo-controlled, phase 2 trial	Monk PD et al	2020	Interferon beta-1	101	57.1	59.18%	Therapeutic	Mar–May 2020
6	Safety and efficacy of the ChAdOx1 nCoV-19 vaccine (AZD1222) against SARS-CoV-2: an interim analysis of four randomised controlled trials in Brazil, South Africa, and the UK	Voysey M et al	2021	ChAdOx1 nCoV-19 (AZD1222)	7548		38.69%	Vaccine	Apr–Nov 2020
7	Efficacy of ChAdOx1 nCoV-19 (AZD1222) vaccine against SARS-CoV-2 variant of concern 202,012/01 (B.1.1.7): an exploratory analysis of a randomised controlled trial	Emary KRW et al	2021	ChAdOx1 nCoV-19 (AZD1222)	8534		40.65%	Vaccine	May–Nov 2020
8	Lopinavir-ritonavir in patients admitted to hospital with COVID-19 (RECOVERY): a randomised, controlled, open-label, platform trial	Horby PW et al	2020	Lopinavir-ritonavir	5040	66.3	61.05%	Therapeutic	Mar–Jun 2020
9	Inhaled budesonide in the treatment of early COVID-19 (STOIC): a phase 2, open-label, randomised controlled trial	Ramakrishnan S et al	2021	Budesonide	139	45	42.45%	Therapeutic	Jul–Dec 2020
10	Convalescent plasma in patients admitted to hospital with COVID-19 (RECOVERY): a randomised controlled, open-label, platform trial	RECOVERY collaborative	2021	Convalescent plasma	11,558	63.5	64.28%	Therapeutic	May 2020–Jan 2021
11	Tocilizumab in patients admitted to hospital with COVID-19 (RECOVERY): a randomised, controlled, open-label, platform trial	RECOVERY collaborative	2021	Tocilizumab	4116	63.6	67.40%	Therapeutic	Apr 2020–Jan 2021
12	Human safety, tolerability, and pharmacokinetics of molnupiravir, a novel broad-spectrum oral antiviral agent with activity against SARS-CoV-2	Painter WP et al	2021	Molnupiravir	130	38.7	83.85%	Therapeutic	
13	Azithromycin in patients admitted to hospital with COVID-19 (RECOVERY): a randomised, controlled, open-label, platform trial	RECOVERY collaborative	2021	Azithromycin	7763	65.2	62.08%	Therapeutic	Apr–Nov 2020
14	Single-dose administration and the influence of the timing of the booster dose on immunogenicity and efficacy of ChAdOx1 nCoV-19 (AZD1222) vaccine: a pooled analysis of four randomised trials	Voysey M et al	2021	ChAdOx1 nCoV-19 (AZD1222)	8948		40.87%	Vaccine	Apr–Dec 2020
15	Safety and immunogenicity of ChAdOx1 nCoV-19 vaccine administered in a prime-boost regimen in young and old adults (COV002): a single-blind, randomised, controlled, phase 2/3 trial	Ramasamy MN et al	2020	ChAdOx1 nCoV-19 (COV002)	552	60.5	50.18%	Vaccine	May–Aug 2020
16	Safety and immunogenicity of the ChadOx1 nCoV-19 vaccine against SARS-CoV-2: a preliminary report of a phase 1/2, single-blind, randomised controlled trial	Folegatti PM et al	2020	ChadOx1 nCoV-19	1077	35	50.23%	Vaccine	Apr–May 2020
17	Dexamethasone in Hospitalized Patients with Covid-19	RECOVERY collaborative	2021	Dexamethasone	6425	66.1	63.37%	Therapeutic	Mar–June 2020
18	Effect of Hydroxychloroquine in Hospitalized Patients with COVID-19: Preliminary results from a multi-centre, randomized, controlled trial	RECOVERY collaborative	2020	Hydroxychloro- quine	4716	65.4	62.21%	Therapeutic	Mar–June 2020
19	Effect of noninvasive respiratory strategies on intubation or mortality among patients with acute hypoxaemic respiratory failure and COVID-19: The RECOVERY-RS randomized clinical trial	Perkins G et al	2022	Non-invasive ventilatory strategies	1273	56.7	66.30%	Therapeutic	Apr 2020–May 2021
20	An online breathing and wellbeing programme (ENO Breathe) for people with persistent symptoms following COVID-19: a parallel group, single blind, randomised controlled trial	Philip KEJ et al	2022	Online breathing and wellbeing programme	150	49.5	17.69%	Therapeutic	Apr–May 2021
21	Colchicine for Covid-19 in the community (PRINCIPLE): a randomised, controlled, adaptive platform trial	Dorward J et al	2022	Colchicine	1381	61		Therapeutic	Mar–May 2021
22	Inspiratory muscle training enhances recovery post COVID-19: a randomised clinical trial	McNarry MA et al	2022	Inspiratory muscles	148	46	11.49%	Therapeutic	
23	Casirivimab and imdevimab in patients admitted to hospital with COVID-19 (RECOVERY): a randomised, controlled open-label, platform trial	RECOVERY collaborative	2022	Casirivimab and imdevimab	9785		62.63%	Therapeutic	Sep 2020–May 2021
24	Namilumab or inflixmab compared with standard of care in hospitalised patients with COVID-19 (CATALYST): a randomised, multicentre, multi-arm, multistage, open-label, adaptive, phase 2, proof of concept trial	Fisher BA et al	2022	Namilumab or inflixmab	146	58.4	61.64%	Therapeutic	Jun 2020–Feb 2021
25	Immunogenicity, safety, and reactogenicity of heterologous COVID-19 primary vaccination incorporating mRNA, viral-vector and protein-adjuvant vaccines in the UK (Com-COV2): a single blind, randomised, phase 2, non-inferiority trial	Stuart ASV et al	2022	mRNA, viral-vector and protein-adjuvant vaccines	532	62	39.47%	Vaccine	Apr–May 2021
26	Safety and immunogenicity of seven COVID-19 vaccines as a third dose (booster) following two doses of ChAdOx1 nCov-19 or BNT162b2 in the UK (COV-BOOST): a blinded, multicentre, randomised, controlled, phase 2 trial	Munro APS et al	2021	ChAdOx1 nCov-19 or BNT162b2	2883		49.84%	Vaccine	Jun 2021
27	Safety and immunogenicity of heterologous versus homologous prime-boost schedules with adenoviral vectored and mRNA COVID-19 vaccine (Com-COV): a single-blind, randomised, non-inferiority trial	Liu X et al	2021	heterologous v homologous prime-boost with adenoviral vectored and mRNA COVID-19 vaccine	463	57.8	54.21%	Vaccine	Feb 2021
28	Azithromycin versus standard care in patients with mild-to-moderate COVID-19 (ATOMIC2): an open-label, randomised trial	Hinks TSC et al	2021	Azithromycin	295	45.9	51.53%	Therapeutic	Jun 2020–Jan 2021
29	Favipiravir, lopinavir-ritonavir or combination therapy (FLARE): a randomised, double blind, 2 × 2 factorial placebo-controlled trial of early antiviral therapy in COVID-19	Lowe DM et al	2022	Favipiravir, lopinavir-ritonavir or combination therapy	240	40	51.25%	Therapeutic	Oct 2020–Nov 2021
30	Aspirin in patients admitted to hospital with COVID-19 (RECOVERY): a randomised, controlled, open-label, platform trial	RECOVERY collaborative	2022	Aspirin	14,892	59.2	61.78%	Therapeutic	Nov 2020–Mar 2021

The total number of patients included in the meta-analysis was 118,912. The mean age of participants was 61.1 (range 35–66.3) years, and 55.03% (range 11.49–83.85%) were male. The trials enrolled patients between March 2020 and November 2021.

Reporting of ethnicity through different stages of the trial was limited and inconsistent (Table 3). Of the 30 trials in the review, none reported data on those approached for inclusion by ethnicity. No trials reported data on those screened for inclusion by individual ethnicity, though seven trials, all from the RECOVERY (Randomised Evaluation of COVID-19 Therapy) group (https://www.recoverytrial.net/), reported these data as “White”, “BAME” (Black, Asian and minority ethnic) or “Unknown”, which was not further disaggregated. The percentage of “White” participants was under-represented in all 7 trials compared with ONS population statistics at this stage, with approximately a quarter of participants documented as “BAME” or “Unknown” in all trials (Table 4). Similarly, no trials reported data on those either deemed eligible for inclusion or deemed ineligible for inclusion by individual ethnicity, though the same seven trials from the RECOVERY group reported these data as “White”, “BAME” or “Unknown”. The proportion of those deemed eligible was similar to that at the screening stage, while the proportion of those deemed ineligible compared to the screening stage was more varied, with no clear pattern indicating whether “White” or “BAME” participants were more likely to be ineligible.

**Table 3:** A summary of the reporting by ethnicity through different stages of COVID-19 clinical trials, including those approached, screened and deemed eligible or ineligible for inclusion; those enrolled in the trial; those followed up at the primary end point and longest follow-up; and reporting of effect estimates

Stage of trial	No. of trials reporting by ethnicity (no. participants)	References	No. of trials reporting as “White”, “BAME”, “non-white”, “Other” (no. participants)	References
Approached for inclusion	0		0	
Screened for inclusion	0		7 (60,179)	[8, 10, 13, 17, 18, 23, 26]
Eligible for inclusion	0		7 (60,179)	[8, 10, 13, 17, 18, 23, 26]
Ineligible for inclusion	0		7 (60,179)	[8, 10, 13, 17, 18, 23, 26]
Enrolled in trial	17 (52,747)	[1–4, 6, 7, 12, 14–16, 19, 20, 24, 25, 27–29]	11 (64,722)	[5, 8–11, 13, 17, 18, 23, 26, 30]
Followed up at primary endpoint	0		9 (64,434)	[8–11, 13, 17, 18, 23, 26]
Followed up at longest follow-up	0		0	
Effect estimates	0		11 (79,740)	[1, 8–11, 13, 17–19, 23, 26]

**Table 4:** A summary of the RECOVERY group’s reporting of ethnicity at different trial stages, including those approached for inclusion; those deemed eligible or ineligible for inclusion; and those followed up at the primary endpoint. The RECOVERY trials reported ethnicity as “White”, “BAME” or “non-white”, and did not disaggregate this data further

Trial no	No. of people approached for inclusion reported by ethnicity			No. of people deemed eligible for inclusion reported by ethnicity			No. of people deemed ineligible for inclusion reported by ethnicity				No. of people followed up at primary endpoint reported by ethnicity		
	**White (%)**	**BAME (%)**	**Unknown (%)**	**White (%)**	**BAME (%)**	**Unknown (%)**	**White (%)**	**BAME (%)**	**Unknown (%)**		**White (%)**	**BAME (%)**	**Unknown (%)**
**8**	6060 (75)	1351 (17)	692 (9)	3781 (75)	865 (17)	394 (8)	2279 (74)	486 (16)		298 (10)	3781	865	
**10**	10,810 (75)	2271 (16)	1241 (9)	8914 (77)	1720 (15)	924 (8)	1896 (69)	551 (20)		317 (11)	8914	1720	
**11**											3127 (76)	732 (18)	257 (6)
**13**	9301 (75)	1852 (15)	1180 (10)	5939 (77)	1109 (14)	715 (9)	3362 (74)	743 (16)		465 (10)	5939	1109	
**17**	6015 (74)	1420 (17)	697 (9)	4689 (73)	1147 (18)	589 (9)	1326 (78)	273 (16)		108 (6)	4689 (73)	1147 (18)	589 (9)
**18**	5860 (73)	1365 (18)	690 (9)	3479 (74)	857 (18)	380 (8)	2381 (74)	508 (16)		310 (10)	3479	857	
**23**	10,039 (77)	1809 (14)	1184 (9)	7601 (78)	1293 (13)	891 (9)	2438 (75)	516 (16)		293 (9)	7601	1293	
**30**	16,189 (76)	3260 (15)	1900 (9)	11,129 (75)	2378 (16)	1385 (9)	5690 (80)	882 (12)		515 (7)	11,129	2378	

Seventeen trials reported the number of people enrolled in the trial by individual ethnicity, though one of these only reported participants as “White”, “Black” or “Other”. A further eleven grouped enrolled participants into “White” vs “BAME”, “White” vs “non-white” or “White” vs “Other” (in this case meaning all non-White participants). The percentage of those enrolled in these studies is discussed in detail in the meta-analysis. In the seven RECOVERY trials for which there is complete data, there were no loss of participants from those deemed eligible for inclusion and subsequently enrolled in the study.

None of the trials reported those followed up at the primary endpoint by individual ethnicity, though eight trials, all from the RECOVERY group, reported this data as “White”, “BAME” or “Unknown”, with none reporting loss to follow-up from those enrolled in the trial. One further trial reported the primary outcome for White participants only. No trials reported by ethnicity at the longest follow-up, though all the RECOVERY trials made reference to “further analyses specified at 6 months”. None of the trials reported effect estimates by ethnicity, though eleven trials report this data for “White”, “BAME” or “Other” groups, and do not disaggregate this further.

Three studies documented specific strategies to improve recruitment of ethnic minority groups, and three studies mentioned recruiting from ethnic minority communities, though no details were provided (Table 5). None of these trials recruited a higher proportion of participants from ethnic minority communities compared to those not reporting recruitment strategies.

**Table 5:** A summary of the strategies used to improve recruitment of ethnic minority groups in COVID-19 trials

Trial no	Strategies to improve recruitment
2. Yu LM er al	“To increase recruitment from ethnic minority and socially deprived communities, which have been disproportionately affected by COVID-19, we used several outreach strategies, including the appointment in September, 2020, of an expert working with ethnic minorities; active collaboration with community, religious and health organisations; and promotion in multiple languages through a range of media.”
4. Butler CC et al	“Given the increased risk from COVID-19 among Black, Asian, and minority ethnic communities, we actively reached out to a range of religious and community organisations at national and regional levels to increase participation from diverse backgrounds.”
19. Perkins G et al.*	“Collection and reporting of race and ethnicity based on fixed categories and mandated by funder due to disproportionate effect of COVID-19 infection on non-white population.” – no specific recruitment strategy described
21. Doward J et al	“Several community outreach strategies were implemented aiming to increase recruitment of those from ethnically diverse communities and socioeconomically deprived backgrounds, who have been disproportionally affected by COVID-19.”
26. Munro APS et al.*	“Recruitment of those identifying as black or minority ethnic was particularly encouraged.”—no specific recruitment strategy described

**Fig. 2:**
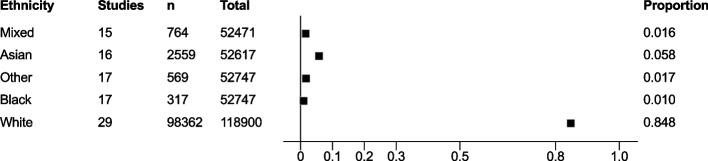
Summary of meta-analysis of enrolment to trials

**Fig. 3:**
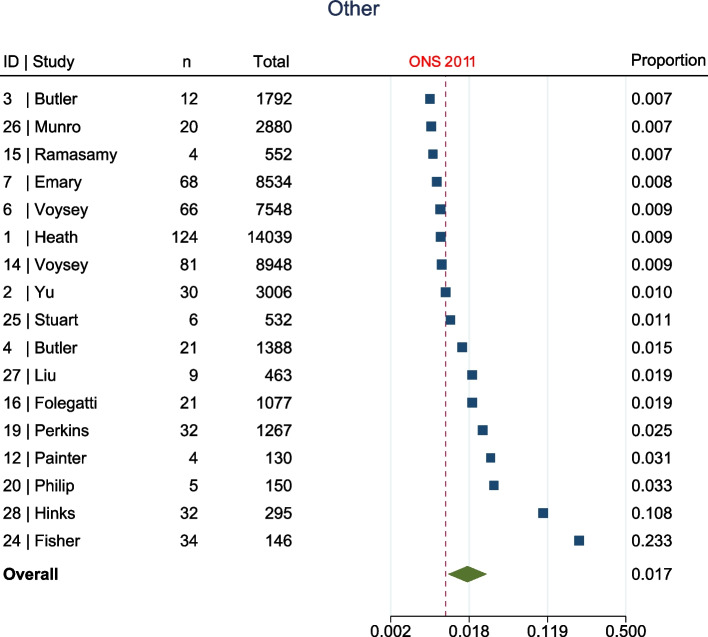
Seventeen trials documented enrolment of Other participants. Overall effect shows Other participants were over-represented when compared to ONS statistics (1.7% [95% CI 1.1–2.8%] vs ONS 1%)

**Fig. 4:**
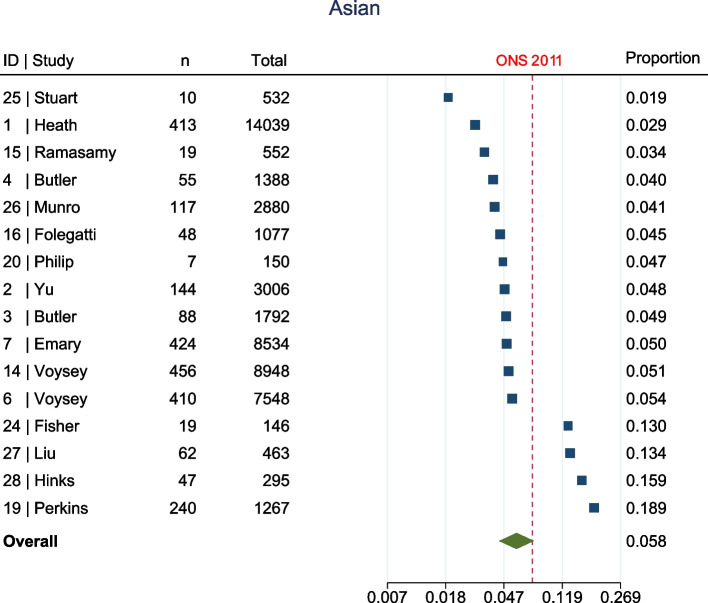
Sixteen trials documented enrolment of Asian participants. Overall effect shows Asian participants were under-represented when compared to ONS statistics (5.8% [95% CI 4.4–7.6%] vs ONS 7.5%)

**Fig. 5:**
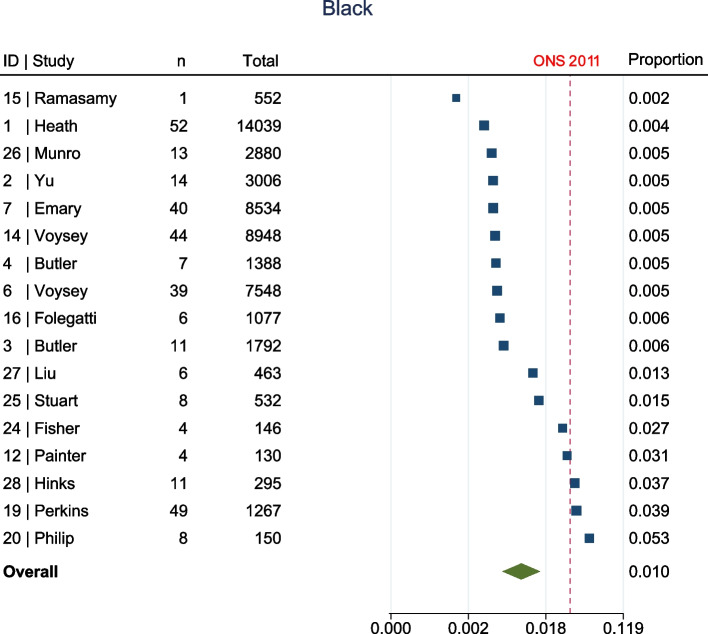
Seventeen trials documented enrolment of Black participants. Overall effect shows Black participants were under-represented when compared to ONS statistics (1% [95% CI 0.6–1.5%] vs ONS 3.3%)

**Fig. 6:**
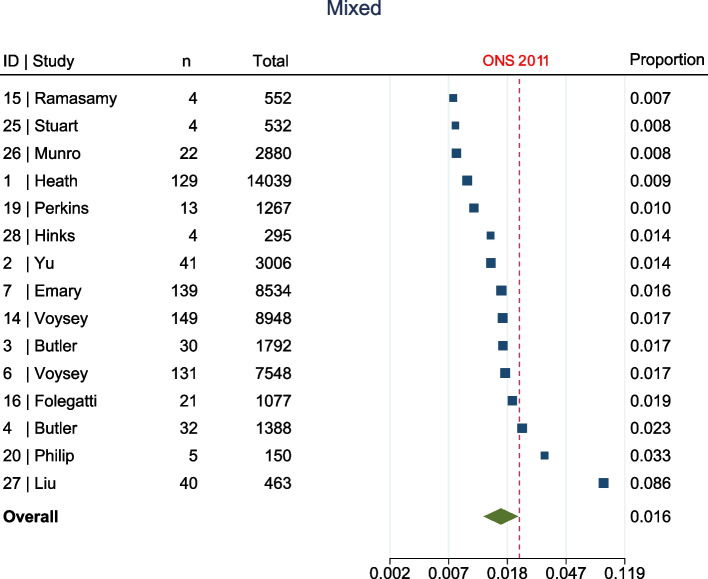
Fifteen trials documented enrolment of Mixed participants. Overall effect shows Mixed participants were under-represented when compared to ONS statistics (1.6% [95% CI1.2–2.1%] vs ONS 2.2%)

**Fig. 7:**
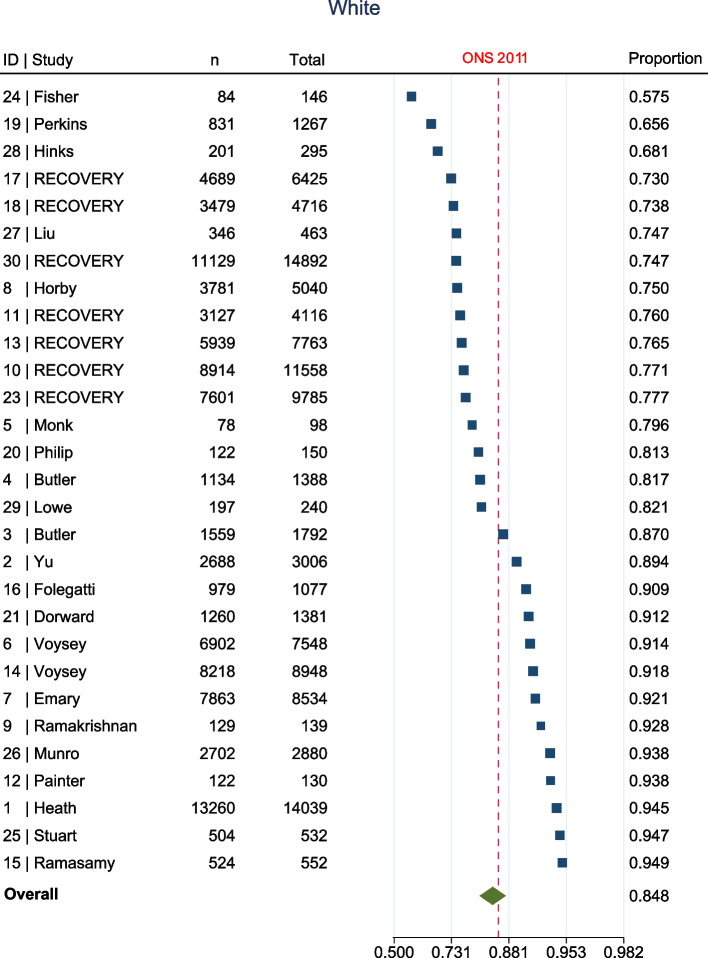
Twenty-nine trials documented enrolment of White participants. Overall effect shows White participants were under-represented when compared to ONS statistics (84.8% [95% CI 81.6–87.5%] vs ONS 86%)

The meta-analysis, summarised in Fig. 2, shows that at enrolment to the trial, all ethnic groups, apart from Other (1.7% [95% confidence interval (CI) 1.1–2.8%] vs ONS 1%, Fig. 3), were represented to a lesser extent than that suggested by 2011 ONS statistics. This was most marked in the Asian (5.8% [95% CI 4.4–7.6%] vs ONS 7.5%, Fig. 4) and Black (1% [95% CI 0.6–1.5%] vs ONS 3.3%, Fig. 5) groups, though also apparent in the Mixed (1.6% [95% CI1.2–2.1%] vs ONS 2.2%, Fig. 6) and White (84.8% [95% CI 81.6–87.5%] vs ONS 86%, Fig. 7) ethnic groups. The meta-analysis shows significant variation across studies, in relation to census-expected proportions of patients recruited from different ethnic groups.

**Fig. 8:**
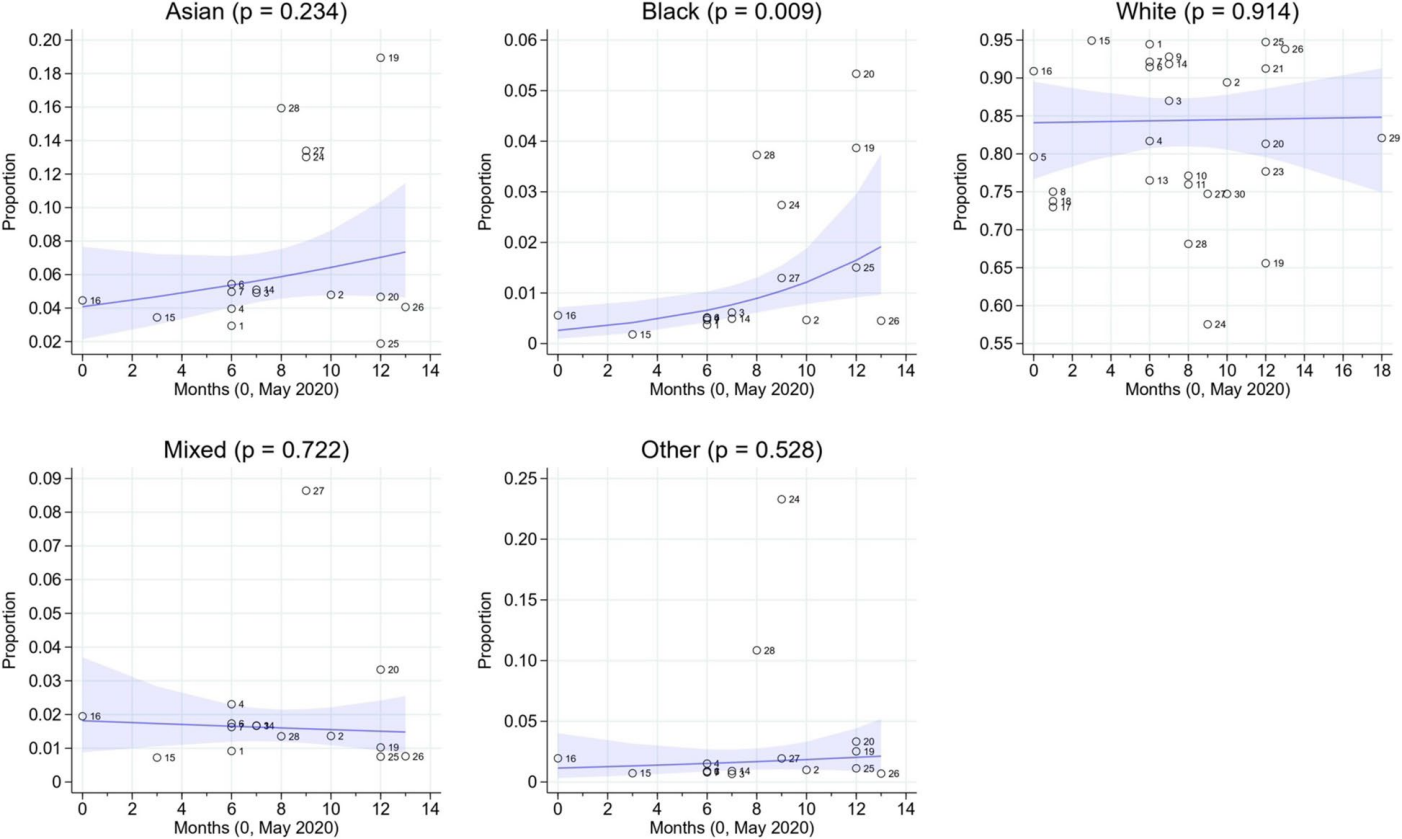
Results of the meta-regression. Recruitment in the Black ethnic group improved from May 2020 to June 2021 (from an estimated 0.26% to 1.92%, *p* = 0.009). There were no statistically significant temporal trends in the other groups

Figure 8 illustrates the results of the meta-regression, which shows that recruitment in the Black ethnic group improved from May 2020 to June 2021 (from an estimated 0.26 to 1.92%, *p* = 0.009). There were no statistically significant temporal trends in the other groups (Asian (*p* = 0.234), White (*p* = 0.914), Other (*p* = 0.528) and Mixed (*p* = 0.722).

## Discussion

We conducted an extensive review of literature to determine any disparities in the representation of ethnic minority groups in UK COVID-19 clinical trials. Our meta-analysis findings demonstrate that in 30 trials of over 100,000 participants, the Asian, Black, Mixed and White ethnic groups were represented to a lesser extent than that suggested by 2011 ONS statistics. There is significant heterogeneity in the proportions of participants recruited from different ethnic groups across studies, though the Asian and Black groups demonstrate the greatest proportion of studies below the percentages demonstrated by the 2011 census. These results might indicate one of two things: first, that Asian and Black groups were enrolled at lower percentages than population averages in more studies than White or Other groups, though whether this interpretation withstands scrutiny is unclear, as the White ethnic group were also not over-represented in the majority of the data. This may lead us to a second conclusion: that Asian, Black and Mixed ethnicities were more likely to be classified as “Other”, grouped into problematic “non-White” or “BAME” categories, or not recorded at all (for example, as “Unknown”).

If the first interpretation is correct, it suggests that ethnic minority groups are more likely to be under-represented in COVID-19 trials. This continues a trend of poor recruitment from European trials when compared to North American trials, though neither have shown a temporal improvement in representation of ethnic minority participants [27]. There is also a large body of evidence which has identified previous racial and ethnic enrolment disparities in other types of medical research including trials on cancer, diabetes [20, 28] and cardiovascular disease [29] over the last decade. This may be particularly concerning as the hospital population during the pandemic did not reflect ONS population statistics, with a higher proportion of inpatients from ethnic minority communities [30], in theory providing a larger pool for research teams to recruit from. This raises the important consideration of what population triallists should aspire to map to. Should trial recruitment aim to be representative of the general population, or of the population to whom the interventions are most relevant? If the latter, we might expect vaccine trials, which are designed for whole populations, to map to ONS data, but to use a different reference point for treatments for severe disease which affect a greater proportion of ethnic minority groups, such as tocilizumab for severe COVID-19 infection.

If the second interpretation is true, it raises questions about data accuracy and reporting. There is a distinct lack of consistency in the reporting of results by ethnicity, with many studies continuing to use the term “BAME”, which is no longer favoured due to its emphasis on certain ethnic groups, to the exclusion of others [26]. Harmonisation of data is made difficult by differences in ethnicity coding internationally [31], and there have been calls for more detailed and consistent ethnicity coding [32]. Moreover, one could legitimately question the utility of presenting results for genetically, phenotypically, and culturally heterogenous groups under one umbrella (“BAME” or “non-white”), and indeed the authors suggest that in many cases this is to bolster numbers, increasing the likelihood that subgroup analyses are statistically significant.

It is important to clarify why all groups, apart from “Other” appear to be under-represented in the data. The weighted averages do not include individuals grouped under terms such as “BAME”, “non-White” or “Other” (referencing non-White), as this disaggregated data was not available. Therefore, all groups appear to be under-represented as a proportion of the total.

The meta-regression showed that over the course of the pandemic, recruitment in the Black group improved over the study period (*p* = 0.009), while no significant temporal trends were seen in the other ethnic groups. Improved recruitment amongst the Black ethnicity could be due to the recognition that COVID-19 disproportionately affected ethnic minorities, leading to calls to increase and encourage recruitment from these communities [33], as illustrated in Table 5.

Recruitment to trials, however, is far from the only issue. Our findings show limited reporting by ethnicity at all stages. Enrolment to trials was best reported, with seventeen of the 30 studies breaking participants down by individual ethnic groups. The meta-analysis highlighted that while twenty-nine reported participant enrolment for the White ethnicity, only 17, 16 and 15 studies reported participant enrolment for the Black, Asian and Mixed ethnicity groups respectively. Some of the other studies grouped individuals from minority communities as “BAME” and in these studies “BAME” representation was higher than UK ONS data (if including Asian, Black, Mixed and Other groups from ONS statistics, though such definitions in these studies were not always clear). It is important to highlight that no information was available on the representation of individual ethnicities within these studies, or indeed the “non-White” grouping used by other studies. The over-representation of this grouping of ethnicities is in direct contrast to the studies that reported enrolment data by individual ethnic groups, where a lower proportion of Black, Asian and Mixed participants were enrolled when compared to ONS data in the majority of the studies (14/17, 12/16 and 12/15, respectively).

Understanding enrolment disparities and data absenteeism in RCTs is vital as a lack of diversity can bias the results and limit generalisability to underrepresented populations. It is acknowledged that genetic polymorphisms can affect responses to vaccines [34] and medicinal therapeutics, such as antihypertensives, heart failure medications and warfarin [35]. Interventions which have been predominantly tested in White populations may not be as effective in other ethnicities [20, 36], and indeed a lack of representation in trials for vaccines and therapeutics fuels mistrust and vaccine hesitancy amongst minority communities [22].

A variety of reasons for the underrepresentation of ethnic minorities have been proposed. These can broadly be grouped into three categories: those occurring at system, individual and interpersonal levels. Barriers at the healthcare system and hospital level include restrictive study designs, financial costs associated with running trials and lack of community engagement. Commonly reported individual barriers revolve around lack of comfort, lack of knowledge on the research process and the study, logistics and time and resource constraints [37]. Doctor-patient relationships, including a lack of support if problems arose [22], language barriers [23], and mistrust (particularly suspicions of a hidden agenda [22]) play an important role at an interpersonal level [38, 39]. Overcoming these barriers is key to improving recruitment and participation in medical research, and tailored strategies will need to be implemented to improve participation in research of ethnic minority groups. A one-size fits all approach is inadequate as barriers identified vary between community groups [22]. These issues need to be approached at the conceptualisation of a trial, and inclusion of ethnic minorities should be considered at all stages of the research process [23].

Previous studies have shown that community-based [40, 41] and multimedia interventions [42] can be effective in increasing participation in research. Despite this, we found only three studies designed specific strategies to improve recruitment of ethnic minority groups (Table 5). None of these trials recruited a higher proportion of participants from ethnic minority communities, suggesting the issue requires a more complex solution. Two further studies made mention of recruiting from Black, Asian or minority ethnic communities, one mandated by the funder; however, no details were made available for how this was pursued.

The Consolidated Standard of Reporting Trials (CONSORT) statement provides guidance to researchers on reporting findings from RCTs. It advocates providing baseline demographic and clinical features of participants [43]. However, it does not specify which sociodemographic characteristics should be captured or presented. In order to combat underrepresentation, the National Institute for Health and Care Research recently developed the Innovations in Clinical Trial Design and Delivery for the Under-served (INCLUDE) framework, a tool that provides specific guidance to researchers on improving recruitment of minority groups [44]. Such an approach appears to have borne fruit in North America, where the Food and Drug Administration (FDA)-published guidance emphasises collecting racial and ethnic data in clinical trials, and appears to have improved the collection of such data [27].

In addition to using such frameworks, we call for consistency in the reporting of ethnicity in randomised controlled trials; first, in the terminology used to describe ethnic groups, both in the UK and internationally, to enable comparison of data across trials [45]. Second, to avoid grouping multiple ethnic groups under one term, ensuring better reporting of effect estimates by different ethnic groups. And third, collecting and reporting ethnicity data at every stage of a trial, increasing transparency and improving our understanding of where failures to recruit or retain participants occur.

Our study had several strengths. Our search strategy was robust, and our data collection methods were piloted and rigorous. Each full-text article was reviewed by two authors. We performed a meta-analysis and meta-regression, which provided a better estimate of the percentage and improved the generalisability of our findings. Limitations include that our systematic review assessed only UK-based studies, thus our findings are not generalisable to other countries. However, this was to allow comparison of our findings with high-quality data on proportion of patients from different ethnic groups in our national datasets. Second, some multinational studies that recruited from UK centres did not break down their results by individual country, meaning these were excluded, which may have biased the results. Third, the least biased pooled estimate for the analyses was for the White ethnicity, which was available for all studies; for other ethnicities, missing data may have impacted on the pooled estimate (for example, where ethnicities have been grouped into “BAME” rather than reported separately). Fourth, the population statistics we used were based on 2011 ONS data, which may not reflect today’s population statistics. Data from the 2021 census is awaited, but it is likely that changes to the demographic composition of the UK in this time will shine a harsher light on poor representation from ethnic minority communities. A further sub-analysis of interest is an examination of the characteristics of the organisation hosting the trial, and the region where the study took place, as there is significant heterogeneity in ethnic diversity in the UK. However, many of the studies we included were national or multi-site and did not disaggregate their results by region or site, making such an analysis impossible. This represents an area for future research. Finally, we have used one approach to the analysis of proportions, while others are available.

## Conclusions

This systematic review of 30 trials with over 100,000 participants shows that Asian, Black and Mixed ethnic groups are either under-represented to a greater extent or incorrectly documented as “BAME”, “Other”, “non-white” or “Unknown” in UK COVID-19 RCTs. Underrepresentation in clinical trials occur at the system, individual and interpersonal level and require complex solutions, which should be approached at trial conception and considered throughout the research process. Reporting of trials by ethnicity lacks consistency, with obsolete terminology in common use, and grouping of multiple ethnicities commonplace despite genetic and phenotypic differences. Few trials report specific methods for recruiting participants from ethnic minorities. Those conducting trials need to make use of available frameworks for recruiting patients and report data that are consistent in terminology and have greater transparency.

### Supplementary Information

**Additional file 1**: **Appendix 1.** Search strategy. **Appendix 2.** Numbers used for the analysis. **Appendix 3.** PRISMA abstract checklist. **Appendix 4.** PRISMA checklist. **Appendix 5.** PRISMA 2020 flow diagram for new systematic reviews which included searches of databases and registries only.

## Data Availability

Data collected for this study and the study protocol will be made available with publication of the manuscript and can be obtained by emailing the corresponding author, after investigator support is obtained.

## Abbreviations

ARC-EM: Applied Research Collaboration East Midlands
BAME: Black, Asian and minority ethnic
CI: Confidence interval
CONSORT: Consolidated Standards of Reporting Trials
COVID-19: Coronavirus disease
FDA: Food and Drug Administration
INCLUDE: Innovations in Clinical Trial Design and Delivery for the Under-served
mRNA: Messenger ribonucleic acid
NIHR: National Institute for Health and Care Research
ONS: Office for National Statistics
PRISMA: Preferred Reporting Items for Systematic Review and Meta-Analyses
PROSPERO: International Prospective Register of Systematic Reviews
RECOVERY: Randomised Evaluation of COVID-19 Therapy
RCT: Randomised controlled trial
SARS-CoV-2: Severe acute respiratory distress syndrome 2
UK: United Kingdom
