# Supplementary material for: Ethnic minority representation in UK COVID-19 trials: systematic review and meta-analysis
Source: BMC Med. 2023 Mar 29;21:111. doi: 10.1186/s12916-023-02809-7 (PMC10049782; doi:10.1186/s12916-023-02809-7)
Supplement: Supplementary file 1 — Additional file 1: Appendix 1. Search strategy. Appendix 2. Numbers used for the analysis. Appendix 3. PRISMA abstract checklist. Appendix 4. PRISMA checklist. Appendix 5. PRISMA 2020 flow diagram for new systematic reviews which included searches of databases and registries only. [file 12916_2023_2809_MOESM1_ESM.docx]

## **Supplementary Materials**

**Appendix 1 – search strategy**

**NICE search terms for COVID-19 for MEDLINE (Ovid)**

Adapted from Levay and Finnegan (2021). The NICE COVID-19 search strategy for Ovid MEDLINE and EMBASE: developing and maintaining a strategy to support rapid guidelines. NICE. Figure 4. Doi: https://doi.org/10.1101/2021.06.11.21258749

1 SARS-CoV-2/ or COVID-19/

2 (corona* adj1 (virus* or viral*)).ti,ab,kw,kf.

3 (CoV not (Coefficien* or "co-efficien*" or covalent* or Covington* or covariant* or covarianc* or "cut-off value*" or "cutoff value*" or "cut-off volume*" or "cutoff volume*" or "combined optimi?ation value*" or "central vessel trunk*" or CoVR or CoVS)).ti,ab,kw,kf.

4 (coronavirus* or 2019nCoV* or 19nCoV* or "2019 novel*" or Ncov* or "n-cov" or "SARSCoV-2*" or "SARSCoV-2*" or SARSCoV2* or "SARS-CoV2*" or "severe acute respiratory syndrome*" or COVID*2).ti,ab,kw,kf.

5 or/1-4

6 limit 5 to yr="2020-Current"

7 (6 and english.lg.) not (letter or historical article or comment or editorial or news).pt. not (Animals/ not humans/)

**UK search terms for MEDLINE (Ovid)**

Adapted from Ayiku et al (2020). The NICE UK geographic search filters for MEDLINE and Embase (Ovid): post-development study to further evaluate precision and number-needed-to-read when retrieving UK evidence. *Research Synthesis Methods* 11 (5): 669-677. doi: [**https://doi.org/10.1002/jrsm.1431**](https://doi.org/10.1002/jrsm.1431)

1 exp United Kingdom/

2 (national health service* or nhs*).ti,ab,in.

3 (english not ((published or publication* or translat* or written or language* or speak* or literature or citation*) adj5 english)).ti,ab.

4 (gb or "g.b." or britain* or (british* not "british columbia") or uk or "u.k." or united kingdom* or (england* not "new england") or northern ireland* or northern irish* or scotland* or scottish* or ((wales or "south wales") not "new south wales") or welsh*).ti,ab,jw,in.

5 (bangor or "bangor's" or cardiff or "cardiff's" or newport or "newport's " or st asaph or "st asaph's" or st davids or swansea or "swansea's").ti,ab,in.

6 (aberdeen or "aberdeen's" or dundee or "dundee's" or edinburgh or "edinburgh's" or glasgow or "glasgow's" or inverness or (perth not australia*) or ("perth's" not australia*) or stirling or "stirling's").ti,ab,in.

7 (armagh or "armagh's" or belfast or "belfast's" or lisburn or "lisburn's" or londonderry or "londonderry's" or derry or "derry's" or newry or "newry's").ti,ab,in.

8 (bath or "bath's" or ((Birmingham not alabama*) or ("birmingham's" not alabama*) or bradford or "bradford's" or brighton or "brighton's" or bristol or "bristol's" or carlisle* or "carlisle's" or (cambridge not (massachusetts* or boston* or harvard*)) or ("cambridge's" not (massachusetts* or boston* or harvard*)) or (canterbury not zealand*) or ("canterbury's" not zealand*) or chelmsford or "chelmsford's" or chester or "chester's" or chichester or "chichester's" or coventry or "coventry's" or derby or "derby's" or (durham not (carolina* or nc)) or ("durham's" not (carolina* or nc)) or ely or "ely's" or exeter or "exeter's" or gloucester or "gloucester's" or hereford or "hereford's" or hull or "hull's" or lancaster or "lancaster's" or leeds* or leicester or "leicester's" or (lincoln not nebraska*) or ("lincoln's" not nebraska*) or (liverpool not (new south wales* or nsw)) or ("liverpool's" not (new south wales* or nsw)) or ((london not (ontario* or ont or toronto*)) or ("london's" not (ontario* or ont or toronto*)) or manchester or "manchester's" or (newcastle not (new south wales* or nsw)) or ("newcastle's" not (new south wales* or nsw)) or norwich or "norwich's" or nottingham or "nottingham's" or oxford or "oxford's" or peterborough or "peterborough's" or plymouth or "plymouth's" or portsmouth or "portsmouth's" or preston or "preston's" or ripon or "ripon's" or salford or "salford's" or salisbury or "salisbury's" or sheffield or "sheffield's" or southampton or "southampton's" or st albans or stoke or "stoke's" or sunderland or "sunderland's" or truro or "truro's" or wakefield or "wakefield's" or wells or westminster or "westminster's" or winchester or "winchester's" or wolverhampton or "wolverhampton's" or (worcester not (massachusetts* or boston* or harvard*)) or ("worcester's" not (massachuse tts* or boston* or harvard*)) or (york not ("new york*" or ny or ontario* or ont or toronto*)) or ("york's" not ("new york*" or ny or ontario* or ont or toronto*))))).ti,ab,in.

9 2 or 3 or 4 or 5 or 6 or 7 or 8 or 9

10 (exp africa/ or exp americas/ or exp antarctic regions/ or exp arctic regions/ or exp asia/ or exp oceania/) not (exp great britain/ or europe/)

11 10 not 11

12 1 and 12

**Search terms for RCTs:**

1 randomized controlled [trial.pt](http://trial.pt/).

2 controlled clinical [trial.pt](http://trial.pt/).

3 randomized.ab.

4 placebo.ab.

5 drug therapy.fs.

6 randomly.ab.

7 trial.ab.

8 groups.ab.

9 1 or 2 or 3 or 4 or 5 or 6 or 7 or 8

10 exp animals/ not humans.sh.

11 9 not 10

**Google Scholar search terms:**

The following search terms were used until we found 3 consecutive pages with no relevant results.

(covid OR coronavirus OR sars) (trial OR trials OR rct OR rcts) (UK OR "united kingdom" OR england OR english OR scotland OR scottish) (britain OR british OR wales OR welsh OR ireland OR irish)

**Appendix 2 – Numbers used for the analysis**

Numbers used for the analyses

| **No** | **name** | **Asian** | **Black** | **White** | **Mixed** | **Other** | **Unknown** | **Total** | **time** |
| --- | --- | --- | --- | --- | --- | --- | --- | --- | --- |
| 1 | 1 \| Heath | 413 | 52 | 13260 | 129 | 124 | 61 | 14039 | Nov-20 |
| 2 | 2 \| Yu | 144 | 14 | 2688 | 41 | 30 | 89 | 3006 | Mar-21 |
| 3 | 3 \| Butler | 88 | 11 | 1559 | 30 | 12 | 92 | 1792 | Dec-20 |
| 4 | 4 \| Butler | 55 | 7 | 1134 | 32 | 21 | 139 | 1388 | Nov-20 |
| 5 | 5 \| Monk |  |  | 78 |  |  |  | 98 | May-20 |
| 6 | 6 \| Voysey | 410 | 39 | 6902 | 131 | 66 | 0 | 7548 | Nov-20 |
| 7 | 7 \| Emary | 424 | 40 | 7863 | 139 | 68 | 0 | 8534 | Nov-20 |
| 8 | 8 \| Horby |  |  | 3781 |  |  | 394 | 5040 | Jun-20 |
| 9 | 9 \| Ramakrishnan |  |  | 129 |  |  |  | 139 | Dec-20 |
| 10 | 10 \| RECOVERY |  |  | 8914 |  |  | 924 | 11558 | Jan-21 |
| 11 | 11 \| RECOVERY |  |  | 3127 |  |  | 257 | 4116 | Jan-21 |
| 12 | 12 \| Painter | 0 | 4 | 122 | 0 | 4 | 0 | 130 |  |
| 13 | 13 \| RECOVERY |  |  | 5939 |  |  | 715 | 7763 | Nov-20 |
| 14 | 14 \| Voysey | 456 | 44 | 8218 | 149 | 81 | 0 | 8948 | Dec-20 |
| 15 | 15 \| Ramasamy | 19 | 1 | 524 | 4 | 4 | 0 | 552 | Aug-20 |
| 16 | 16 \| Folegatti | 48 | 6 | 979 | 21 | 21 | 2 | 1077 | May-20 |
| 17 | 17 \| RECOVERY |  |  | 4689 | 0 | 0 | 589 | 6425 | Jun-20 |
| 18 | 18 \| RECOVERY |  |  | 3479 | 0 | 0 | 380 | 4716 | Jun-20 |
| 19 | 19 \| Perkins | 240 | 49 | 831 | 13 | 32 | 102 | 1267 | May-21 |
| 20 | 20 \| Philip | 7 | 8 | 122 | 5 | 5 | 3 | 150 | May-21 |
| 21 | 21 \| Dorward |  |  | 1260 |  |  |  | 1381 | May-21 |
| 23 | 23 \| RECOVERY |  |  | 7601 |  |  | 891 | 9785 | May-21 |
| 24 | 24 \| Fisher | 19 | 4 | 84 | 0 | 34 | 5 | 146 | Feb-21 |
| 25 | 25 \| Stuart | 10 | 8 | 504 | 4 | 6 | 0 | 532 | May-21 |
| 26 | 26 \| Munro | 117 | 13 | 2702 | 22 | 20 | 6 | 2880 | Jun-21 |
| 27 | 27 \| Liu | 62 | 6 | 346 | 40 | 9 | 0 | 463 | Feb-21 |
| 28 | 28 \| Hinks | 47 | 11 | 201 | 4 | 32 | 0 | 295 | Jan-21 |
| 29 | 29 \| Lowe |  |  | 197 |  |  |  | 240 | Nov-21 |
| 30 | 30 \| RECOVERY |  |  | 11129 |  |  | 1385 | 14892 | Mar-21 |

This table shows that the least biased estimation is for the White ethnic group (available for all studies), while some studies had missing data for other ethnicities, which may have impacted on the pooled estimate (for example, for RECOVERY 17, the total is 6425 participants, but the sum 4689+589=5278, indicating that there are people of other ethnic groups which have not been reported, in this case documented as “BAME”).

**Appendix 3: PRISMA abstract checklist**

| **Section and Topic** | **Item #** | **Checklist item** | **Reported (Yes/No)** |
| --- | --- | --- | --- |
| **TITLE** | | |  |
| Title | 1 | Identify the report as a systematic review. | Yes |
| **BACKGROUND** | | |  |
| Objectives | 2 | Provide an explicit statement of the main objective(s) or question(s) the review addresses. | Yes |
| **METHODS** | | |  |
| Eligibility criteria | 3 | Specify the inclusion and exclusion criteria for the review. | Yes |
| Information sources | 4 | Specify the information sources (e.g. databases, registers) used to identify studies and the date when each was last searched. | Yes |
| Risk of bias | 5 | Specify the methods used to assess risk of bias in the included studies. | Yes |
| Synthesis of results | 6 | Specify the methods used to present and synthesise results. | Yes |
| **RESULTS** | | |  |
| Included studies | 7 | Give the total number of included studies and participants and summarise relevant characteristics of studies. | Yes |
| Synthesis of results | 8 | Present results for main outcomes, preferably indicating the number of included studies and participants for each. If meta-analysis was done, report the summary estimate and confidence/credible interval. If comparing groups, indicate the direction of the effect (i.e. which group is favoured). | Yes |
| **DISCUSSION** | | |  |
| Limitations of evidence | 9 | Provide a brief summary of the limitations of the evidence included in the review (e.g. study risk of bias, inconsistency and imprecision). | Yes |
| Interpretation | 10 | Provide a general interpretation of the results and important implications. | Yes |
| **OTHER** | | |  |
| Funding | 11 | Specify the primary source of funding for the review. | N/A |
| Registration | 12 | Provide the register name and registration number. | Yes |

**Appendix 4: PRISMA checklist**

| **Section and Topic** | **Item #** | **Checklist item** | **Location where item is reported** |
| --- | --- | --- | --- |
| **TITLE** | | |  |
| Title | 1 | Identify the report as a systematic review. | Page 1 |
| **ABSTRACT** | | |  |
| Abstract | 2 | See the PRISMA 2020 for Abstracts checklist. | 2-3 |
| **INTRODUCTION** | | |  |
| Rationale | 3 | Describe the rationale for the review in the context of existing knowledge. | 4 |
| Objectives | 4 | Provide an explicit statement of the objective(s) or question(s) the review addresses. | 5 |
| **METHODS** | | |  |
| Eligibility criteria | 5 | Specify the inclusion and exclusion criteria for the review and how studies were grouped for the syntheses. | 6, Table 1 |
| Information sources | 6 | Specify all databases, registers, websites, organisations, reference lists and other sources searched or consulted to identify studies. Specify the date when each source was last searched or consulted. | 6-7, Supplementary Materials |
| Search strategy | 7 | Present the full search strategies for all databases, registers and websites, including any filters and limits used. | Supplementary Materials |
| Selection process | 8 | Specify the methods used to decide whether a study met the inclusion criteria of the review, including how many reviewers screened each record and each report retrieved, whether they worked independently, and if applicable, details of automation tools used in the process. | 6-7  Figure 1 |
| Data collection process | 9 | Specify the methods used to collect data from reports, including how many reviewers collected data from each report, whether they worked independently, any processes for obtaining or confirming data from study investigators, and if applicable, details of automation tools used in the process. | 7 |
| Data items | 10a | List and define all outcomes for which data were sought. Specify whether all results that were compatible with each outcome domain in each study were sought (e.g. for all measures, time points, analyses), and if not, the methods used to decide which results to collect. | 7-8 |
|  | 10b | List and define all other variables for which data were sought (e.g. participant and intervention characteristics, funding sources). Describe any assumptions made about any missing or unclear information. | 7-8 |
| Study risk of bias assessment | 11 | Specify the methods used to assess risk of bias in the included studies, including details of the tool(s) used, how many reviewers assessed each study and whether they worked independently, and if applicable, details of automation tools used in the process. | N/A |
| Effect measures | 12 | Specify for each outcome the effect measure(s) (e.g. risk ratio, mean difference) used in the synthesis or presentation of results. | 8 |
| Synthesis methods | 13a | Describe the processes used to decide which studies were eligible for each synthesis (e.g. tabulating the study intervention characteristics and comparing against the planned groups for each synthesis (item #5)). | 6  Table 1 |
|  | 13b | Describe any methods required to prepare the data for presentation or synthesis, such as handling of missing summary statistics, or data conversions. | 8 |
|  | 13c | Describe any methods used to tabulate or visually display results of individual studies and syntheses. | 8 |
|  | 13d | Describe any methods used to synthesize results and provide a rationale for the choice(s). If meta-analysis was performed, describe the model(s), method(s) to identify the presence and extent of statistical heterogeneity, and software package(s) used. | 8 |
|  | 13e | Describe any methods used to explore possible causes of heterogeneity among study results (e.g. subgroup analysis, meta-regression). | 8 |
|  | 13f | Describe any sensitivity analyses conducted to assess robustness of the synthesized results. | N/A |
| Reporting bias assessment | 14 | Describe any methods used to assess risk of bias due to missing results in a synthesis (arising from reporting biases). | N/A |
| Certainty assessment | 15 | Describe any methods used to assess certainty (or confidence) in the body of evidence for an outcome. | 8 |
| **RESULTS** | | |  |
| Study selection | 16a | Describe the results of the search and selection process, from the number of records identified in the search to the number of studies included in the review, ideally using a flow diagram. | 10  Figure 1 |
|  | 16b | Cite studies that might appear to meet the inclusion criteria, but which were excluded, and explain why they were excluded. | N/A |
| Study characteristics | 17 | Cite each included study and present its characteristics. | Table 2 |
| Risk of bias in studies | 18 | Present assessments of risk of bias for each included study. | N/A |
| Results of individual studies | 19 | For all outcomes, present, for each study: (a) summary statistics for each group (where appropriate) and (b) an effect estimate and its precision (e.g. confidence/credible interval), ideally using structured tables or plots. | 10-12  Tables 3-5 |
| Results of syntheses | 20a | For each synthesis, briefly summarise the characteristics and risk of bias among contributing studies. | N/A |
|  | 20b | Present results of all statistical syntheses conducted. If meta-analysis was done, present for each the summary estimate and its precision (e.g. confidence/credible interval) and measures of statistical heterogeneity. If comparing groups, describe the direction of the effect. | 12  Figures 2-8 |
|  | 20c | Present results of all investigations of possible causes of heterogeneity among study results. | 12  Figure 2-8 |
|  | 20d | Present results of all sensitivity analyses conducted to assess the robustness of the synthesized results. | 12  Figure 2-8 |
| Reporting biases | 21 | Present assessments of risk of bias due to missing results (arising from reporting biases) for each synthesis assessed. | N/A |
| Certainty of evidence | 22 | Present assessments of certainty (or confidence) in the body of evidence for each outcome assessed. | 12  Yes |
| **DISCUSSION** | | |  |
| Discussion | 23a | Provide a general interpretation of the results in the context of other evidence. | 13-19 |
|  | 23b | Discuss any limitations of the evidence included in the review. | 18-19 |
|  | 23c | Discuss any limitations of the review processes used. | 18-19 |
|  | 23d | Discuss implications of the results for practice, policy, and future research. | 16-18, 20 |
| **OTHER INFORMATION** | | |  |
| Registration and protocol | 24a | Provide registration information for the review, including register name and registration number, or state that the review was not registered. | 2, 6 |
|  | 24b | Indicate where the review protocol can be accessed, or state that a protocol was not prepared. | 6 |
|  | 24c | Describe and explain any amendments to information provided at registration or in the protocol. | 6-8 |
| Support | 25 | Describe sources of financial or non-financial support for the review, and the role of the funders or sponsors in the review. | 3, 8-9, 23 |
| Competing interests | 26 | Declare any competing interests of review authors. | 22-23 |
| Availability of data, code and other materials | 27 | Report which of the following are publicly available and where they can be found: template data collection forms; data extracted from included studies; data used for all analyses; analytic code; any other materials used in the review. | N/A |

**Appendix 5: PRISMA 2020 flow diagram for new systematic reviews which included searches of databases and registries only**

**Identification of studies via databases and registers**

Records removed *before screening*:

(n = 0)

Records identified from:

MEDLINE (Ovid) (n = 4,139)

Google Scholar (n = 1,000)

**Identification**

Records screened

(n = 5,319)

Records excluded

(n = 5,096)

Reports sought for retrieval

(n = 223)

Reports not retrieved

(n = 0)

**Screening**

Reports excluded:

Not vaccine or therapeutic (n = 50)

Did not disaggregate UK data (n = 33)

Not an RCT (n = 32)

Duplication (n = 32)

Not a UK study (n = 28)

Sample size < 50 (n = 10)

Participants < 18 (n = 8)

Reports assessed for eligibility

(n = 223)

Studies included in review

(n = 30)

**Included**
